# Fabrication of fluorescent nanospheres by heating PEGylated tetratyrosine nanofibers

**DOI:** 10.1038/s41598-020-79396-7

**Published:** 2021-01-28

**Authors:** Enrico Gallo, Carlo Diaferia, Nicole Balasco, Teresa Sibillano, Valentina Roviello, Cinzia Giannini, Luigi Vitagliano, Giancarlo Morelli, Antonella Accardo

**Affiliations:** 1 IRCCS SDN, Via E. Gianturco 113, 80143 Naples, Italy; 2 Department of Pharmacy, Research Centre on Bioactive Peptides (CIRPeB), University of Naples “Federico II”, Via Mezzocannone 16, 80134 Naples, Italy; 3 Institute of Biostructures and Bioimaging (IBB), CNR, Via Mezzocannone 16, 80134 Naples, Italy; 4 Institute of Crystallography (IC), CNR, Via Amendola 122, 70126 Bari, Italy; 5 Department of Chemical, Materials and Industrial Production Engineering, DICMaPI, University of Naples “Federico II”, Piazzale V. Tecchio 80, 80125 Naples, Italy

**Keywords:** Chemistry, Materials science, Nanoscience and technology

## Abstract

Aromatic polypeptides have recently drawn the interest of the research community for their capability to self-assemble into a variety of functional nanostructures. Due to their interesting mechanical, electrical and optical properties, these nanostructures have been proposed as innovative materials in different biomedical, biotechnological and industrial fields. Recently, several efforts have been employed in the development of these innovative materials as nanoscale fluorescence (FL) imaging probes. In this context, we describe the synthesis and the functional properties of a novel fluorescent tyrosine (Tyr, Y)-based nanospheres, obtained by heating at 200 °C a solution of the PEGylated tetra-peptide PEG6-Y4. At room temperature, this peptide self-assembles into not fluorescent low ordered water-soluble fibrillary aggregates. After heating, the aggregation of different polyphenolic species generates Y4-based nanospheres able to emit FL into blue, green and red spectral regions, both in solution and at the solid state. The aggregation features of PEG6-Y4 before and after heating were studied using a set of complementary techniques (Fluorescence, CD, FT-IR, Small and Wide-Angle X-ray Scattering and SEM). After a deep investigation of their optoelectronic properties, these nanospheres could be exploited as promising tools for precise biomedicine in advanced nanomedical technologies (local bioimaging, light diagnostics, therapy, optogenetics and health monitoring).

## Introduction

**Figure 1:**
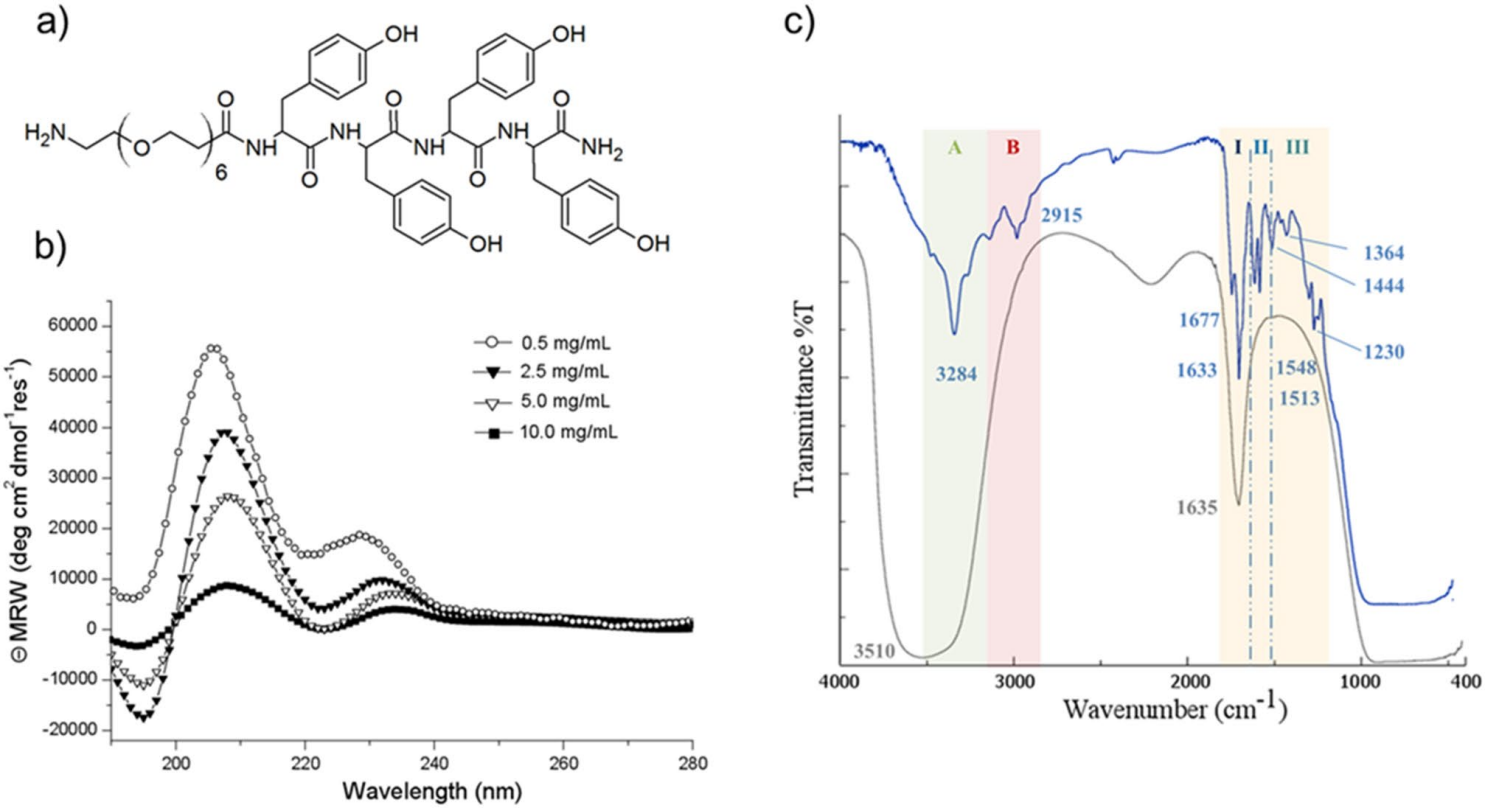
(**a**) Schematic representation of PEG6-Y4. (**b**) CD spectra profiles of PEG6-Y4 recorded in water in 190–280 nm range at several concentrations. (**c**) FTIR spectra of PEG6-Y4 in water solution at a concentration of 2.0 mg/mL (grey line) and at solid state on the sample obtained by drying the sample sin solution (blue line).

In the last years, nanoscale fluorescence imaging has emerged as innovative diagnostic tool for the biomedical research area and for applications in precision medicine^1^. Fluorescence imaging is a powerful technique that could allow getting a deep comprehension of some essential biological processes, including imaging of mechanisms occurring into the living biocells. However, performances of this technique are strictly related to the employed fluorescent marker. A broad range of fluorescent dyes (such as organic molecules^2,3^, carbon dots^4^, semiconductor inorganic quantum dots^5^), able to cover the entire visible spectrum, has been proposed until now. Due to their capability to combine high quantum yield and photostability with high biocompatibility and biodegradability, recently, bioinspired peptide-based nanostructures have been envisioned as powerful fluorescent imaging probes^6–9^. These nano/micro-structures can be generated by spontaneous self-assembly of short and ultrashort aromatic peptides under specific experimental conditions of pH, temperature and solvent media^10–12^. Noteworthy is the example of the diphenylalanine (FF) homopeptide^13^ that aggregates into various nanostructures (nanospheres, fibers, hydrogels, nanoporous framework or Venturi-like nanotubes)^14^. These different supramolecular structures exhibit intriguing mechanical^15^, electrical^16^, electrochemical^17^ and optoelectronic^18–20^ physical properties. In particular, it was observed the appearance of blue/green photoluminescence (PL) in FF nanostructures, upon thermally induced phase^19–21^. A very similar behaviour was also observed for self-assembled nanostructures of LL dipeptide and FFF tripeptide. This fluorescence phenomenon has been observed after heating the sample at temperature ranged between 140–180 °C, as a consequence of an irreversible reconstructive phase transition in the nanostructure^20,21^. Similar optoelectronic properties have been also observed for others amyloid-like fibrils generated by spontaneous self-assembling of several poly-aromatic peptides such as diphenylalanine (F2)^22^, tetra-phenylalanine (F4)^23^, and hexaphenylalanine (F6) derivatized with PEG moieties^24,25^. Self-assembled aggregates of these aromatic peptides share a common structural organization: they assemble into fibrillary nanostructures with a high content of β-sheet structures. Others examples of fluorescent organic nanoparticles via PEGylation of aggregate have been described by Wei’s group as potential bioimaging tools^26,27^. Recently, another example of fluorescent Tyr-nanospheres, emitting blue, green and red light were synthetized by sonochemical approach^28^. In this approach, the cavitation microbubble surface acts as a catalytic binding site to generate polymerization by Tyr-Tyr coupling. The resulting polymerization degree is deeply affected by the ultrasonic frequency, the concentration and time^29^. In the proposed reaction mechanism the formation of phenolic oligomeric species is due to oxidation by OH radicals, that occurs only after π-π stacking interactions between Tyr aromatic rings. Later, ultrasound-assisted methodology was also translated to others aromatic amino acids such as Phe and Trp or to their mixtures^30,31^. In this scenario, here we propose another example of fluorescent Tyr-based nanospheres prepared via dissipative self-assembly by heating the sample. Tyr-nanospheres have been obtained by heating a solution containing the tetra-peptide PEG6-Y4, at 200 °C for 4 h. This polymeric-peptide contains an aromatic framework composed of four Tyr residues and a PEG moiety of six ethoxylic units at its N-terminus (see Fig. 1a). Analogously to the other previously described tetra-peptides (PEG6-F4^32^ and PEG6-W4^33^) and hexa-peptides (PEG8-Y6^34^, PEG8-F6^24^, PEG8-(FY)3^35^), also this Y4-peptide self-assembles in water solution in fibrillary amyloid-like nanostructures. However, as evidences by the ability of fibrillar PEG6-Y4 aggregates to undergo to polymerization at 200 °C leads to the formation of Tyr-based nanospheres that exhibit interesting fluorescence properties that have been here characterized. The application of this strategy led to the obtainment of peptide nanospheres under milder conditions of temperature and pressure compared to those used with ultrasound. Indeed, it was observed that the collapse of a cavitation bubble during the application of the ultrasound leads to the generation of very high temperature and pressure (T > 5000 K and P > 1000 atm, respectively) within the bubble^29^. After a deep investigation of their optoelectronic properties, these nanospheres could be exploited as promising tools for precise biomedicine in advanced nanomedical technologies (local bioimaging, light diagnostics, therapy, optogenetics and health monitoring).

## Results and discussion

### Synthesis and aggregate formulation

PEG6-Y4, whose chemical formula is reported in Fig. 1a, is an aromatic peptide containing four tyrosine residues and a monodisperse polyethylene glycol moiety having six ethoxylic units (PEG6). PEGylation was carried out at the N-terminus of the peptide primary sequence using an Fmoc-protected derivative (Fmoc-Ahoh-OH) directly in solid phase according to the standard Fmoc/tBu protocols^35^. At the end of the synthesis, the peptide was cleaved from the resin, purified by RP-HPLC chromatography and the chemical identity of the product was verified by LC–MS spectrometry and 1HNMR spectroscopy (see Fig. S1). Due to the presence of the PEG moiety and of the phenolic groups on the Tyr side chains, the peptide exhibits a high water solubility (up to 30 mg/mL). This solubility was measured by UV–Vis spectroscopy from the molar extension coefficient at 276 nm, which corresponds to the absorbance wavelength for the tyrosine residue. As previously observed for the other aromatic analogues, PEG6-F4^32^ and PEG6-W4^33^, also PEG6-Y4 is able to self-assemble into supramolecular nanostructures at high concentration. Contrarily to PEG6-F4^32^ and PEG6-W4^33^, whose aggregation occurs upon dilution of a stock peptide solution (100 mg/mL in 1,1,1,3,3,3-hexafluoro-2-propanol, HFIP) in water, PEG6-Y4 self-aggregates directly in water, thus avoiding the use of organic co-solvents. We previously observed the same behavior for PEG8-Y6 analogue^34^. The first evidence of the PEG6-Y4 aggregation was established by the use of the Dynamic Light Scattering (DLS) technique as indicated by the intensity profile of PEG6-Y4 solution at 5.0 mg/mL, reported in Fig. S2a. DLS profile shows a monomodal distribution, thus indicating the presence of one population of aggregates with a mean diameter of ≈ 200 nm.

In order to establish the concentration at which the peptide self-aggregation begins, we tried to measure the critical aggregate concentration (CAC) using a fluorescent method based on the titration with 8-anilinonaphthalene-1-sulfonic acid (ANS). Due to its peculiar spectral features, ANS fluorophore does not emit fluorescence in hydrophilic environments, but only in hydrophobic ones such as the hydrophobic core of assemblies. Contrarily to our expectation, the fluorescence intensity of ANS remains close to zero during the entire titration (data not shown). This unexpected behavior can be attributed to the high water content in the PEG6-Y4 aggregate that avoids an effective fluorophore entrapment.

An estimation of the concentration at which the self-assembly occurs was alternatively obtained by the fluorescence analysis of the Y4-polymer (see Fig. S2b). Taking advantage from the spectroscopic features of the phenol group in the Tyr side chain, emission spectra were acquired at different concentrations, exciting the samples in the absorbance maximum of Tyr at 276 nm. From the inspection of the fluorescence spectra in Fig. S3, a decrease of the emission peak at 303 nm is detectable for samples above 0.4 mg/mL. This quenching, previously reported for other Tyr-containing peptides, is attributable to the aromatic stacking associated with aggregation phenomena of the peptide moiety. According to these data, we can conclude that the aggregation occurs for a peptide concentration between 0.40 and 0.80 mg/mL.

### Secondary structural characterization

Secondary structural organization of the peptide was evaluated by Circular Dichroism (CD) and Fourier Transform Infrared (FTIR) spectroscopies; both techniques are commonly employed for the characterization of peptide-based nanostructures^23^. CD spectra of PEG6-Y4 at several concentrations (ranged between 0.5 and 10 mg/mL) are reported in Fig. 1b. All CD spectra show two maxima at 207 and 228 nm, typically associated with a β-sheet arrangement. Beyond these maxima in the spectra is also detectable a minimum around 195 nm, indicative of the aromatic side-chains stacking occurring as consequence of the β-sheet formation. The maximum at 228 nm undergoes a red shift as function of the concentration that indicates an increase of structuration. The β-sheet arrangement was further confirmed by FTIR spectroscopy. IR spectra of PEG6-Y4 in water solution (grey line) at a concentration of 2.0 mg/mL and at the solid state (blue line) are reported in Fig. 1c. The intense and wide band found in the region between 3000 cm^−1^ and 3700 cm^−1^ for the sample in solution occurs as consequence of the exposure of the aggregate to water that generates asymmetric and symmetric O–H and N–H stretching in the amide A region. Another broad signal, that suggests the occurrence of a β-sheet secondary structure, is clearly detectable in the amide I region centered at 1635 cm^−1^. The spectrum of the sample in its dry form (blue line) is better resolved respect to the spectrum acquired in solution and allows us to obtain major structural information. In details, two principal bands corresponding to amide A (3284 cm^−1^) and amide B (2915 cm^−1^) are detectable. Two different signals in amide I (respectively, 1677 and 1633 cm^−1^) suggest an antiparallel organization of the β-sheets. Moreover, the two bands (located at 1548 and 1513 cm^−1^) in amide II are consequence of the vibrations on the plane of the N–H bond and C-N stretching. Additional C-N stretching vibrations and N–H deformation are responsible for the three bands (1444, 1364 and 1230 cm^−1^) in amide III region.

### Structural characterization at the solid state

#### Wide and small angle X-ray scattering (WAXS/SAXS) and scattering electron microscopy (SEM)

**Figure 2:**
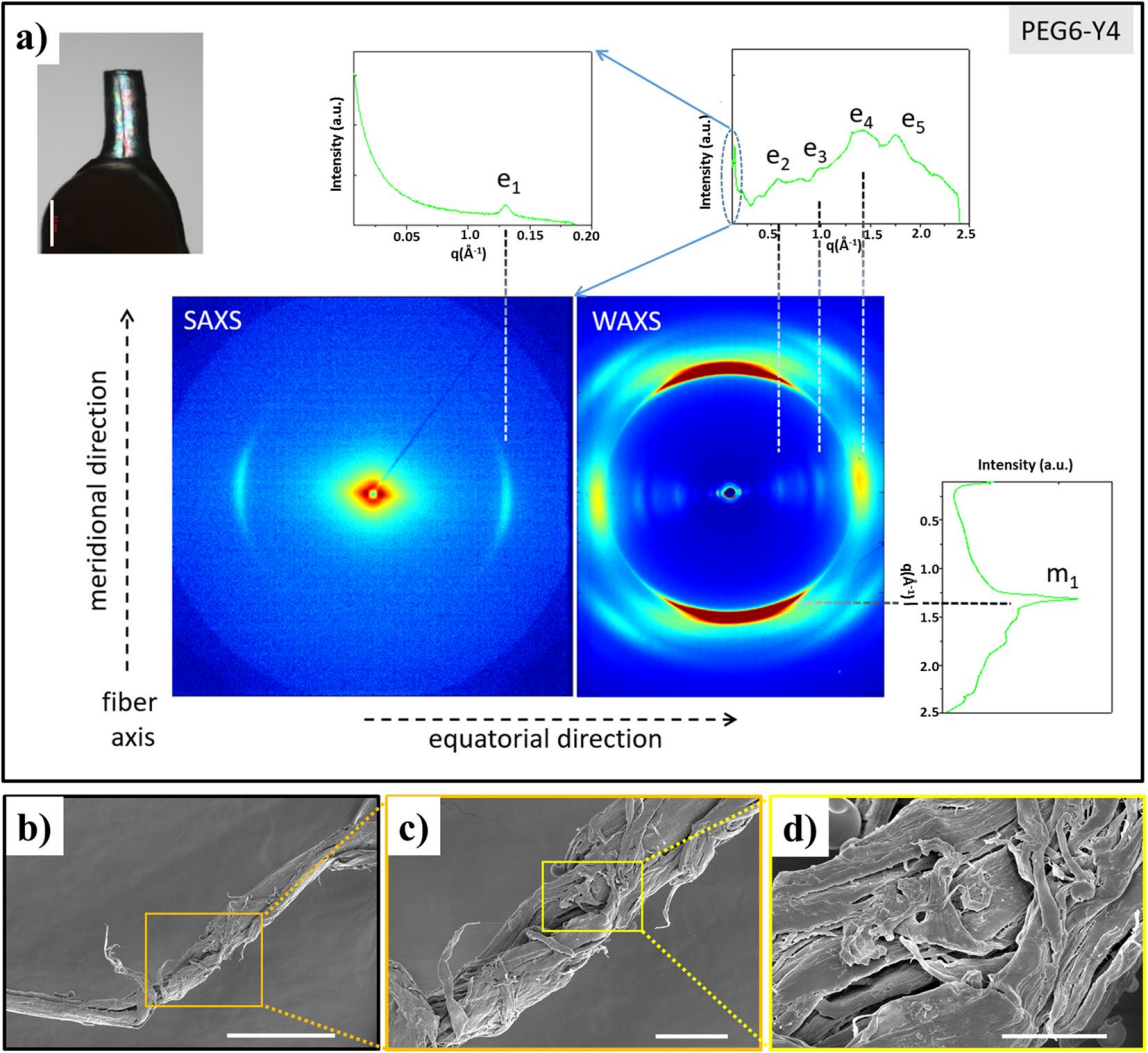
(**a**) Fiber image (white marker corresponds to 0.5 mm), SAXS and WAXS 2D patterns and corresponding 1D profiles integrated along meridional and equatorial directions. (**b**,**c**,**d**) SEM characterization of PEG6-Y4 before heating at different magnifications: (**b**) 3000×, 40 µm scale bar; (**c**) 8000×, 10 µm scale bar and (**d**) 29,000×, 4 µm scale bar.

In order to gain insights into the structural organization of these systems, macroscopic PEG6-Y4 fibers obtained via stretch-frame method^36^ were analyzed in transmission mode by WAXS and SAXS techniques. 2D SAXS and WAXS patterns collected on the fibers highlight the presence of several peaks distributed along two main directions: the meridional direction, which corresponds to the fiber axis, and the equatorial one perpendicular to the first, as shown in Fig. 2a. The 2D patterns were centered, calibrated, and radially folded into 1D profiles. The 1D SAXS profile, is characterized only by a single equatorial peak e1 (q_e1_ = 0.12 Å^−1^; d_e1_ = 52.3 Å). On the other hand, the WAXS pattern presents a rather strong meridional peak m1 (q_m1_ = 1.31 Å^−1^; d_m1_ = 4.8 Å) and a number of minor sharp equatorial peaks [e2 (q_e2_ = 0.57 Å^−1^; d_e2_ = 11 Å), e3 (q_e3_ = 0.98 Å^−1^; d_e3_ = 6.4 Å), e4 (q_e4_ = 1.43 Å^−1^; d_e4_ = 4.4 Å) and e5 (q_e5_ = 1.75 Å^−1^; d_e5_ = 3.6 Å)]. This overall picture is in line with the 2D GIWAXS data collected on a film made by PEG-Y4 fibers (Fig. S3). In this case, the diffracted intensity is anisotropically distributed, mainly along the film surface normal (out-of-plane direction, marked by a black arrow) and perpendicular to it (in-plane direction, marked by a green arrow). The intensity, integrated along the out-of-plane and in-plane directions, shows the same trend of the equatorial and meridional profiles measured by WAXS for the single fiber (see Fig. 2a). In structural terms, the presence of the meridional peak corresponding to a distance of 4.8 Å indicates that the atomic-level structure of PEG6-Y4 fibers, free standing and deposited as film on a substrate, is characterized by β-strands associated in β-sheet elements. The limited sharpness of the equatorial peak at 11 Å is indicative of a rather loose lateral packing of these β-sheets. These structural features of PEG6-Y4 assemblies highlight that PEG6-Y4 assemblies presents a less ordered lateral inter-sheet packing compared to PEG6-Y6 ones^34^. Scanning Electron Microscopy (SEM) measurements provide additional morphological information on the self-assembled PEG6-Y4 nanostructures. Microphotographs acquired on dried samples drop-casted on stub from a 5.0 mg/mL solution point out the formation of fibrillary structures (Fig. 2b-d). These latter nanostructures result from the assembly of smallest fibers with variable length from 2–5 µm up to 50 µm.

#### Molecular dynamics simulations

**Figure 3:**
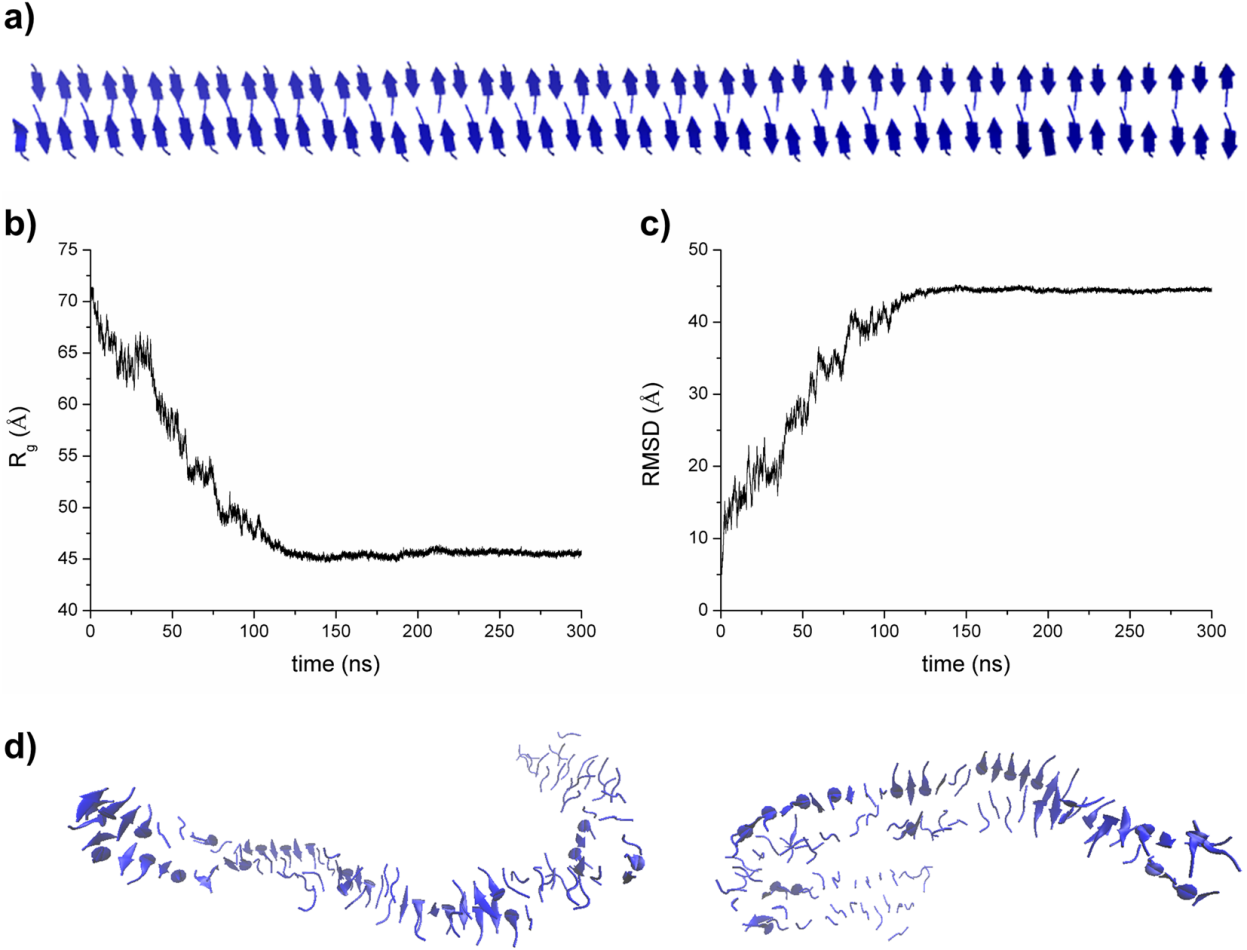
MD simulation of Y4_ST50_SH2. The starting model is reported in panel (**a**). The gyration radius and the RMSD values of the trajectory structures versus the starting flat model are reported in panels (**b**) and (**c**), respectively. Representative examples of trajectory structures are reported in panel (**d**).

In order to gain information on the structure and on the stability of assemblies formed by PEG6-Y4, we performed fully atomistic molecular dynamics (MD) simulations in explicit solvent on multimeric states formed by the Tyr tetrapeptide (Y4). We preliminarily generated models consisting in either a single β-sheet made of fifty strands of four residues (Y4_ST50_SH1) or two closely packed β-sheets generated through the association of a pair of single sheets (Y4_ST50_SH2) (Figs. S4a, 3a). The analysis of the evolution of Y4_ST50_SH1 gyration radius is indicative of a rapid collapse of this model in the early stage of the simulation (Fig. S4b). Accordingly, trajectory structures exhibit high deviations from the starting model (Fig. S4c). The inspection of selected trajectory structures shows major rearrangements of the overall structure of the assembly, with a limited number of regions that locally conserve the β-structure motif. We, then, evaluated the conformational behavior of the two-sheet model in the simulation timescale (300 ns, Fig. 3b). Also in this case, the trajectory structures present significant deviations from the starting model (Fig. 3c), although in this case a significant preservation of the secondary structure is observed. A comparative analysis of these findings with those obtained for the Y6 system^34^ clearly indicates that the reduction of the number of residues in the parent peptide induces a decrease in the stability/rigidity and order of the final assemblies. These observations are in line with the WAXS/SAXS data that indicate less ordered states for the Y4 system compared with the Y6 one previously studied^34^.

#### Heating of PEG6-Y4 assemblies

**Figure 4:**
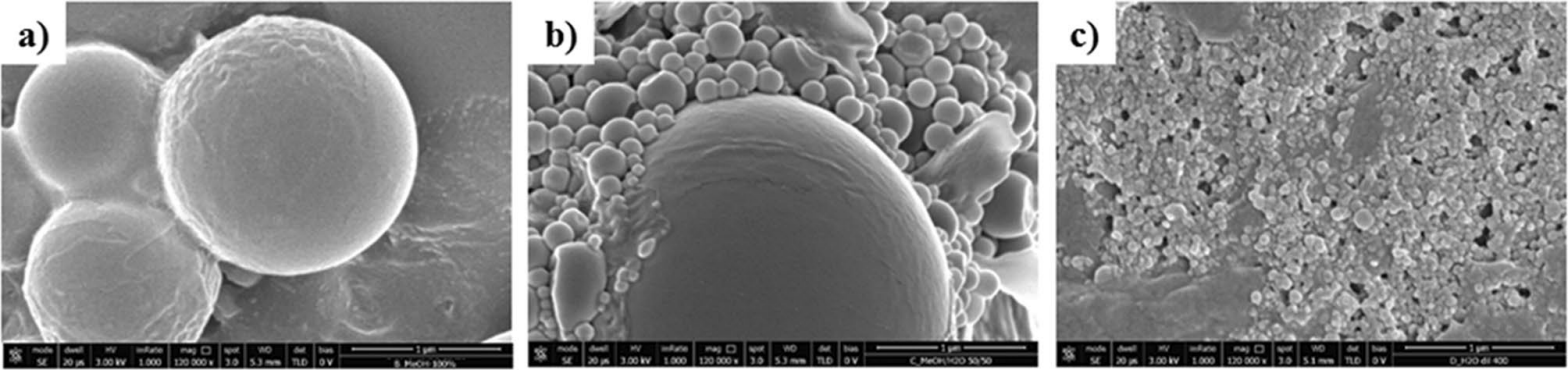
Selected SEM micrographs for self-assembled PEG6-Y4 nanostructures drop-casted after heating in stove for 4 h and re-dissolved in MeOH (**a**), H_2_O/MeOH (50/50, *v/v*) (**b**) and H_2_O/MeOH (0.025/99.75, *v/v*) (**c**). Magnification and scale bar for all the samples are 12,0000×, 1 µm.

Few milliliters of the self-assembled PEG6-Y4 solution at a concentration of 20 mg/mL were heated in stove at 200 °C for different time spans (1, 2 or 4 h). After heating, the peptide powder appears dark brown and exhibits a very low solubility in water with respect to the peptide before heating. Moreover, water solubility decreases proportionally with the time of peptide heat treatments. The reduced solubility can be ascribed to the occurrence of Tyr-Tyr coupling reactions similar to those previously described by Cavalieri et al. that obtained Tyr-based nanospheres with sonochemical approach^28,29^. In this case, the sonication at high frequency ultrasounds of hydrophobic Tyr or its analogues catalyzes the formation of dimeric or polymeric Tyr species. Due to the reduced solubility, thermally treated peptide solutions were prepared in MeOH or in MeOH/H_2_O mixtures (50/50, or 0.25/99.75 *v/v*). CD spectra of the heated peptide in the three solvent conditions are superimposable with spectra before heating, thus indicating the capability of the resulting mixture to keep a β-sheet conformation (data not shown). On the other hand, differently from the peptide at room temperature, heated peptide in MeOH and in H_2_O shows an absorbance maximum around 380 and 420 nm (see Fig. S5). The presence of this additional maximum confirms the formation of novel more conjugated species due to the polymerization. Finally, SEM investigations carried out on the peptide after heating indicate a change in the morphology of the aggregates from fibers to nanospheres (see Figs. 4, S6). SEM microphotographs of the heated peptide after 4 h highlight the presence of nanospheres of different sizes into the three different solvents used for the structural investigation.

In details, there is a significant reduction in the diameter of the spheres as function of the solvent polarity. Indeed, spheres prepared in 100% MeOH have an average size ranged between 500 and 2500 nm, whereas spheres in MeOH/H_2_O have a size of 60–400 nm or 30–150 nm for 50/50 or 0.25/99.75 (*v/v*) mixtures, respectively. In the sample prepared in MeOH/H_2_O (50/50), beyond the smaller nanospheres, we also observe some bigger ones in size. The same trend was also observed for the self-assembled peptide heated for 2 h; but in this instance, in MeOH there is the coexistence of nanospheres and twisted fibers longer than 300 µm (Fig. S6). In order to characterize these nanospheres also in solution, DLS measurements were also performed. Unfortunately, due to the very low concentration of nanoparticles prepared in MeOH/H_2_O 0.25/99.75 (*v/v*), we were not able to record the DLS profile for this sample. As expected, DLS profiles for peptide solutions prepared in pure MeOH and in MeOH/H_2_O (50/50) mixture show a broad monomodal distribution with a mean diameter around 400 and 190 nm, respectively (see Fig. S7). These values are in agreement with the size observed for nanosphers by SEM acquisitions. Although the large size of nanospheres, especially in 100% MeOH, solutions appear clear also after 2 weeks from their preparation and the DLS profiles do not change, thus suggesting a high stability of the peptide nanospheres in the studied conditions (data not shown).

#### Radical scavenger activity (RSA) assay

**Figure 5:**
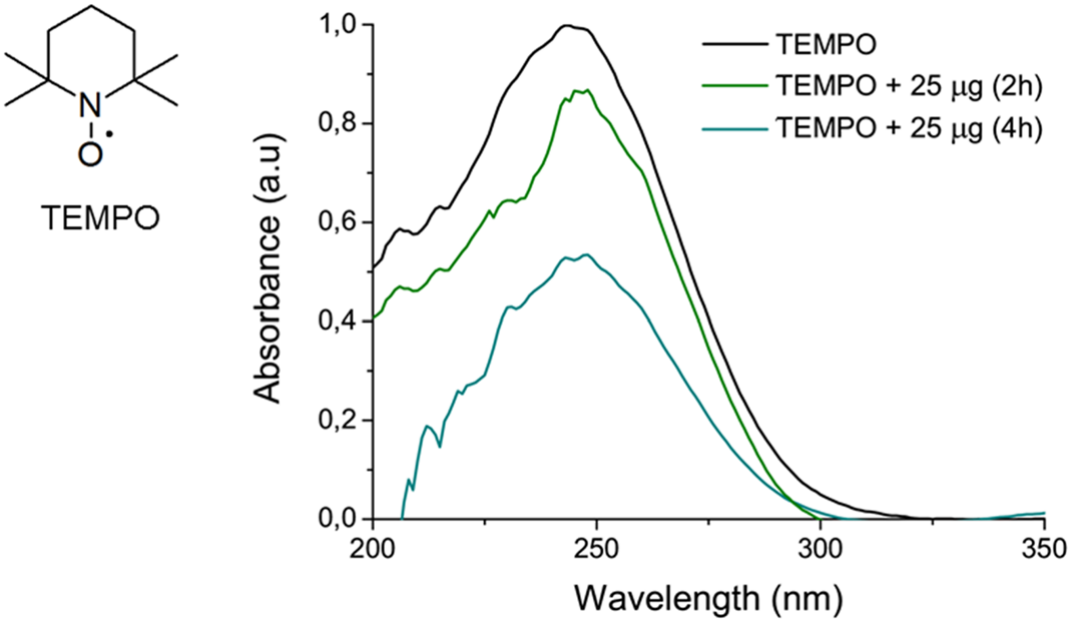
Radical scavenger activity of PEG6-Y4 nanospheres. Comparison of the absorbance UV–Vis spectrum of TEMPO alone and coincubated for 30 min with 25 µg of PEG6-Y4 heated for 2 or 4 h.

Based on the different water solubility of the peptide heated for different time intervals, we can hypothesize the formation of a variable amount of polyphenolic species in the three samples. In order to evaluate the different degree of polymerization of the samples, we estimated their radical scavenger activity (RSA). This latter is related to the deactivation of oxidant species via hydrogen or electron-donation mechanism^37^. For this assay, TEMPO molecule (2,2,6,6-Tetramethyl-1-piperidinyloxy free radical, Fig. 5), which have an absorption maximum around 243 nm, was selected as active radical.

A comparison of the UV–Vis spectrum of the TEMPO solution (at concentration of 5.22 ·10^–4^ mol/L) alone and incubated with 25 μg of the heated species is reported in Fig. 5. From the inspection of the spectra, it can be observed a decrease of the radical absorption in both the heated samples with a RSA percentage of 12% and 49% for the sample treated for 2 and 4 h, respectively. This difference in the RSA could be probably ascribed to the different amount of polyphenolic species in the samples heated for 2 and 4 h. The decrease of the TEMPO absorbance clearly indicates the presence of polyphenolic species in solution. This hypothesis is also supported by the absence of decrease in the negative control in which the TEMPO solution is incubated with the not heated peptide.

#### Fluorescence studies

**Figure 6:**
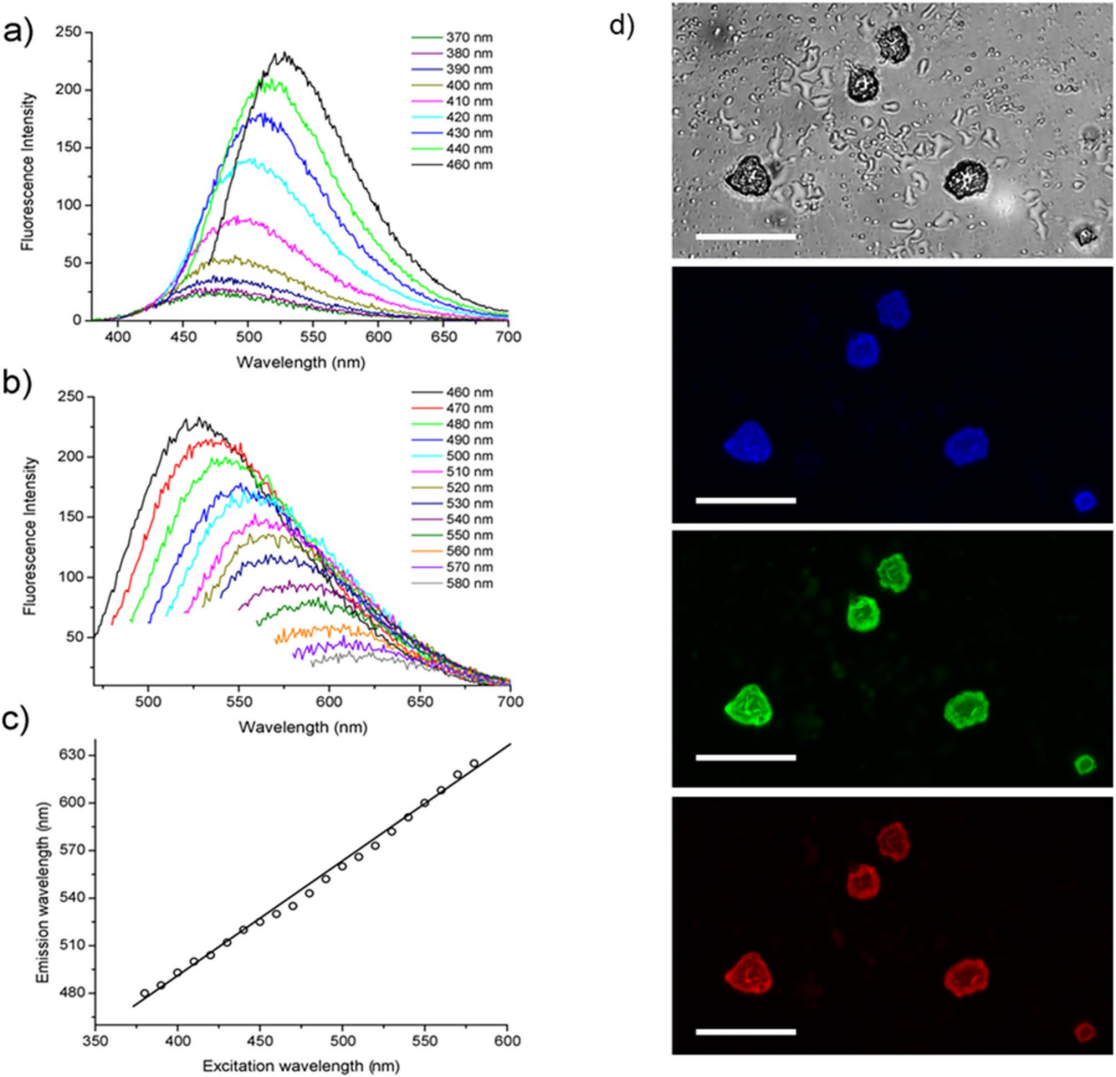
Fluorescence characterization. PL emission spectra of PEG6-Y4 assemblies after 4 h of heating at 5.0 mg/mL in MeOH/H_2_O (50/50) upon excitation in the range (**a**) 370 < λ_exc_ < 460 nm and in the range (**b**) 460 < λ_exc_ < 580 nm. (**c**) Plot of the λ_em_ as function of the λ_exc_. (**d**) Fluorescence microscopy images of the heated peptide solution drop-casted on glass slides and air-dried at room temperature. Images are obtained by exciting the sample in the spectral region of: the bright field; DAPI (4′,6-diamidino-2-phenylindole; λ_exc_ = 359 nm, λ_em_ = 461 nm); GFP (Green Fluorescent Protein; λ_exc_ = 488 nm, λ_em_ = 507 nm) and Rho (Rhodamine; λ_exc_ = 555 nm, λ_em_ = 580 nm). The scale bar of all the images is 5 μm.

The fluorescence behavior of self-assembled PEG6-Y4 nanostructures, before and after heating, was investigated both in solution and at the solid state (Fig. 6). Recently, several studies highlighted the capability of proteins^38,39^ and nanostructures rich in β-sheet^9,24,25,40,41^ to emit an unexpected photoluminescence (PL) in the blue-green visible region (between 440–480 nm) when excited at 370 or 410 nm.

Contrarily to other nanofibrillar structures containing β-sheet, such as fibers of PEG8-F6^24^ or PEG8-Y6^34^, PEG6-Y4 does not emit fluorescence in this spectral region, but exhibits only a peak around 303–305 nm, corresponding to the emission of the Tyr residue. On the other hand, a weak PL can be observed for the sample drop-casted and air-dried on glass (data not shown). The lack of the PL peak at 440–480 nm range for the sample in solution is not surprising considering the structural evidences collected by WAXS and MD simulations on self-assembled PEG6-Y4 that suggest a lower order degree compared to PEG8-Y6^34^ and consequently the occurrence of weak interactions between the peptide side chains in solution. On the other hand, interesting optical and fluorescence properties were found for the peptide assemblies obtained after heating (see Fig. 6). From the inspection of spectra recorded on PEG6-Y4 dissolved in MeOH/H_2_O (50/50) at a concentration of 5.0 mg/mL, it can be observed a red shift of the emission peak by changing the excitation wavelength (Fig. 6a,b). By plotting the λ_em_ as function of the λ_exc_ it can be observed linearity along all the range of wavelengths investigated (Fig. 6c). The spectroscopic behavior observed exciting the sample around 380 nm, which represents the absorbance wavelength of the heated peptide, can be attributed to the presence of several not aggregated fluorescent polymeric Tyr-species in solution. Beyond these peaks, others more intense emission peaks appear in Fig. 6a,b (the maximum at λ_em_ ≈530 nm is observed when the sample is excited at λ_exc_ = 460 nm). Differently from the fluorescence emission peaks due to not aggregated fluorescent polymeric Tyr-species, these additional peaks are originated by the presence in solution of supramolecular nanospheres generated by aromatic interactions between the oligomeric species. A slight red shift for the maximum can be detected for the assemblies prepared in 100% MeOH (Fig. S7). This slight shift is probably attributable to the different polarity of the solvent used for the preparation of the samples. As expected, heated PEG6-Y4 nanospheres keep their optoelectronic properties also at the solid state (Fig. 6d). The same optoelectronic behavior was also observed for the nanospheres prepared by sonochemical approach^28–31^. The photostability of our Tyr-based nanospheres at the solid state was evaluated on the drop-casted sample by comparing the fluorescence bleaching rate of the nanospheres under continuous excitation for 180 min in the GFP spectral region (λexc = 488 nm). Selected immunofluorescence images of nanospheres, recorded at different time points, clearly indicate that the fluorescence intensity of nanoparticles gradually decreased down to half of the initial value after 45 min (Fig. S9). We also evaluated the optoelectronic properties of Tyr-based nanospheres prepared by directly heating the peptide powder in place of the peptide solution. The resulting nanoparticles exhibit the same structural and fluorescence properties (data not shown) of nanoparticles generated in presence of water. This result suggests that contrarily to the sonochemical approach, in which the oxidation by OH radicals is the most dominant mechanism of nanospheres formation, in our preparation method different mechanisms can occur. However, due to the presence of water in traces in air, the oxidation of OH radicals in nanospheres formation mechanism can not be completely ruled out.

## Conclusions

Peptide based building blocks have been recently identified as starting point for the fabrication of novel self-assembled nanomaterials with electrical, magnetic or optoelectronic properties^15–20^. In this scenario, the plethora of short and ultra-short peptides potentially suitable for biotechnological applications has been significantly increased^20–31^. An interesting class of these peptides are the PEGylated peptides, in which the PEG moiety allows increasing the solubility of the resulting material^22,25^. Recently we reported on the synthesis and structural characterization of several PEGylated tetra and hexa-aromatic peptides for applications in tissue engineering and bioimaging. All these peptides self-assemble in water solution with common structural features consisting in their organization into fibrillary nanostructures rich in β-sheet structures with an antiparallel orientation of the β-strands. This secondary structure organization in the peptide assemblies leads to an unexpected photoluminescence in the blue-green spectral regions upon excitation at 370 or 410 nm. Similarly to the previously reported PEGylated aromatic polypeptides, also PEG6-Y4 is able to self-assemble in amyloid like nanostructures. However, a comparative analysis of its structural features with those obtained for the more extended analogue system PEG8-Y6 clearly indicates a decrease of the stability/rigidity and of the order degree of the final assemblies. Due to this lower order into the fibrillary network, PL is not observed in the assemblies prepared in solution, but it appears only in those prepared at the solid state. Nevertheless, we suppose that the intrinsic flexibility, pointed out by the WAXS characterization and MD studies for the Y4 assemblies, promotes the high reactivity of the Tyr residues at high temperature. Indeed, by heating PEG6-Y4 nanostructures at 200 °C we observed a structural transition from twisted fibers to nanospheres. The formation of these latter could be attributed to the polyphenolic species generated at high temperature. Very similar reactions of polymerization were reported for the Tyr residue or Tyr based peptides that have undergone sonication. Here we observe that, analogously to the sonication, the increase of temperature can catalyze Tyr-Tyr coupling reactions with the consequential formation of dimeric or polymeric species. These highly conjugated species exhibit interesting fluorescence properties with a red shift of the emission peak by changing the excitation wavelength.

Our study suggests that the very drastic conditions reached during cavitation bubble (temperature > 5000 K and pressure > 1000 atm)^29^ are not necessary to induce the formation of a fluorescent nanomaterial. Due to their optoelectronic properties Tyr-based nanospheres, easily prepared heating the peptide solution, could be regarded as promising materials to handle as bioimaging agents.

## Materials and methods

Protected N^α^-Fmoc-amino Fmoc-Try(tBu)-OH, coupling reagents and Rink amide MBHA (4-methylbenzhydrylamine) resin were purchased from Calbiochem-Novabiochem (Laufelfingen, Switzerland). The Fmoc-21-amino-4,7,10,13,16,19-hexaoxaheneicosanoic acid [Fmoc-Ahoh-OH, (PEG6)] is commercially available in monodisperse fashion by Neosystem (Strasbourg, France)^33^. All other chemicals were commercially offered by Sigma-Aldrich (Milan, Italy), Fluka (Bucks, Switzerland) or LabScan (Stillorgan, Ireland). They were used as received unless otherwise stated. All solutions were obtained by weight using doubly distilled water as solvent. Preparative RP-HPLCs were carried out on a LC8 Shimadzu HPLC system (Shimadzu Corporation, Kyoto, Japan) equipped with a UV lambda-Max Model 481 detector setting a Phenomenex (Torrance, USA) C_18_ column. Elution solvents are H_2_O/0.1% TFA (A) and CH_3_CN/0.1% TFA (B), from 20 to 70% over 30 min at ϕ = 20 mL/min. Purity and identity of the products were assessed by analytical LC–MS analyses by using Finnigan Surveyor MSQ single quadrupole electrospray ionization (Finnigan/Thermo Electron Corporation San Jose, CA), column: C18-Phenomenex eluted with an H_2_O/0.1% TFA (A) and CH_3_CN/0.1% TFA (B) from 5 to 70% over 15 min at 1 mL/min flow rate^24^.

### Synthesis of the peptide derivative

PEGylated aromatic peptide PEG6-Y4 was synthesized using the solid phase peptide synthesis (SPPS) protocols as previously described^33^. Fmoc/tBu orthogonal strategy was played out. The cleavage from the resin was achieved by treating the peptidyl-resin for 2 h in trifluoroacetic acid (TFA) and using triisopropylsilane (TIS) as scavenger agent. Then, the crude product was precipitated with ice-cold ethyl ether, dissolved in H_2_O/CH_3_CN and freeze-dried. The lyophilized peptide was purified by RP-HPLC chromatography and characterized by ESI mass spectrometry and ^1^H NMR spectroscopy.

PEG6-Y4 characterization: t_R_ = 10.55 min, MS (ESI +): m/z 1005.11 calcd. for C_51_H_66_N_6_O_15_: [M + H^+^] = 1005.72; ^1^H-NMR (CD_3_OD) (chemical shifts in δ, CH_3_OH as internal standard 3.55) = 7.18–7.10 (m, 6 C*H*δ aromatic Tyr), 6.91–6.82 (m, 6 C*H*ε aromatic Tyr), 4.73–4.51 (m, 6H, C*H* α Tyr), 3.87 (s, 16H, OC*H*_*2*_C*H*_*2*_O), 3.82 (t, 8H, RNH-CH_2_C*H*_*2*_O), 3.74–3.71 (m, 8H, OC*H*_*2*_COR), 3.63 (t, 8H, RNH-C*H*_*2*_CH_2_O), 3.31–2.92 (m, 12H, C*H*_*2*_β of Tyr).

### Preparation of peptide solutions

PEG6-Y4 solutions were prepared dissolving the pure peptide powder in water at the desired concentration. The effective peptide concentration in solution was spectroscopically determined by UV–Vis measurements on Thermo Fisher Scientific Inc (Wilmington, Delaware USA) Nanodrop 2000c spectrophotometer equipped with a 1.0 cm quartz cuvette (Hellma) using as molar absorptivity (ε) the value of 5360 M^−1^·cm^−1^^42^.

### Thermal induced phase transition and samples preparation

PEG6-Y4 solution, at a concentration of 20 mg/mL, was heated in oven at 200 °C for 1, 2 or 4 h. The resulting brown powders were resuspended in 100% MeOH or in MeOH/H_2_O mixtures at 50/50 or 0.25/99.75 *v/v*. Briefly, the powder was sonicated in MeOH for 30 min and then centrifuged; the supernatant was directly used or alternatively diluted twofold or 400-fold with water. No precipitation phenomena were detected during the dilution in water.

### Circular Dichroism

Far-UV CD spectra of the peptide in water at several concentrations ranging between 0.50 and 10.0 mg/mL were carried out on a Jasco J-810 spectropolarimeter equipped with a NesLab RTE111 thermal controller unit using a 0.1 mm quartz cell at 25 °C. The spectra were recorded from 280 to 195 nm. Other experimental settings were: scan speed = 10 nm/min; sensitivity = 50 mdeg; time constant = 16 s; bandwidth = 1 nm. Each spectrum was obtained by averaging three scans and corrected for the blank. Here θ represents the mean residue ellipticity (MRE), i.e. the ellipticity per mole of peptide divided by the number of amino acid residues in the peptide^33^.

### Fourier transform infrared spectroscopy (FTIR)

FTIR spectra of the PEGylated peptide derivative in solution (at concentration of 2.0 mg/mL) and at the solid state were collected on a Jasco FT/IR 4100 spectrometer (Easton, MD) in an attenuated total reflection (ATR) mode and using a Ge single-crystal at a resolution of 4 cm^−1^. Each sample was recorded with a total of 100 scans with a rate of 2 mm·s^−1^ against a KBr background. After collection in transmission mode, spectra were converted in emission^33^.

### DLS measurements

Mean diameters of self-assembled nanostructures were measured by Dynamic Light Scattering (DLS) using a Zetasizer Nano ZS (Malvern Instruments, Westborough, MA). Instrumental settings for the measurements are a backscatter detector at 173° in automatic modality, room temperature and disposable sizing cuvette as cell. DLS measurements in triplicate were carried out on aqueous samples at 2.0 mg/mL, after centrifugation at room temperature at 13,000 rpm for 5 min^24^.

### Scanning electron microscopy (SEM)

Morphological analysis of self-assembled peptide nanostructures, before and after heating, was carried out by field emission scanning electron microscope (Nova NanoSem 450-FEI)^22^. Briefly, the sample solutions (5.0 mg/mL), prepared in pure MeOH, or in MeOH/H_2_O mixtures (50/50 or 99.75/0.025, *v/v*), were placed on an aluminium stub using a graphite adhesive tape. A thin coat of gold and palladium was sputtered at a current of 20 mA for 90 s. The sputter coated samples were then introduced into the specimen chamber and the images were acquired at an accelerating voltage of 2–5 kV, spot 3, through the Everhart Thornley Detector (ETD) and the Through the Lens Detector (TLD)^24^.

### Wide-angle and small-angle X-ray scattering

Fiber diffraction WAXS patterns were recorded from dried fibers prepared by the stretch frame method^36^. A droplet (10 μL) of concentrated peptide aqueous solution (~ 1.5 wt%) was suspended between the ends of a wax-coated capillary (spaced 2 mm apart). The droplet was allowed to gently dry at room temperature overnight in order to obtain oriented fibers^24^. The fiber diffraction patterns were collected at the X-ray MicroImaging Laboratory (XMI-L@b) using a set-up equipped with a Fr-E + SuperBright rotating copper anode microsource (45 kV/55 mA; Cu K_α_, λ = 0.15405 nm, 2475 W) coupled through a multilayer focusing optics (Confocal Max-Flux; CMF 15–105) to a three pinholes camera (Rigaku SMAX3000) for Small and Wide Angle X-ray Scattering data collection, also equipped for Grazing Incidence geometry (GISAXS/GIWAXS) by a goniometer with a 125 × 125 mm^2^ stage inserted in the sample chamber^43^. Measurements are performed in vacuum. For WAXS and GIWAXS data collection an 250 × 160 mm^2^ image-plate (IP) detector, with 50 or 100 µm effective pixel size (depending on binning), and off-line RAXIA reader, was inserted at ~ 10 cm (WAXS) and ~ 8.7 cm (GIWAXS) from the sample . For SAXS data collection a second detector, a Triton 20 gas-filled proportional counter (1024 1024 array, 195 mm pixel size) placed at 2.2 m from the sample, was used^44,45^.

### Molecular modelling and dynamics

#### Systems and notations

Three-dimensional models of Y4 aggregates were generated by following the procedure reported in Diaferia et al.^34^ In detail, starting from the hexapeptide fragment KLVFFA of the amyloid-beta peptide II (PDB ID 3OW9) a model composed of a single β-sheet made of fifty strands of four residues was generated (Y4_ST50_SH1) by replacing side chains with Tyr. Considering the organization of the KLVFFA in the crystalline state, a steric zipper model was then generated through the association of a pair of these sheets (Y4_ST50_SH2).

#### Molecular dynamics protocol

MD simulations were carried out on the models we generated (Y4_ST50_SH1 and Y4_ST50_SH2) using the GROMACS software package version 2016.1 (http://manual.gromacs.org/documentation/) with Amber03 force field. These models were immersed in triclinic boxes and solvated with water molecules (TIP3P water model)^46^. The simulations were run applying periodic boundary conditions similar to the conditions previously described F6, (FY)3, Y6 and W4^24,33–35^. Systems were initially energy minimized for 50,000 steps using steepest descent. Then, they were equilibrated for 500 ps at 300 K temperature and for other 500 ps at 1 atm pressure. The Velocity Rescaling and Parrinello-Rahman algorithms were used for pressure and temperature control, respectively. The Particle Mesh Ewald (PME) with a grid spacing of 0.12 nm was used to account for electrostatic interactions. A cut-off of 10 Å was applied to compute the Lennard–Jones interactions. Bond lengths were constrained with the LINCS method. Simulations were run for 100 ns at constant temperature (300 K) and pressure (1 atm) (NpT ensemble) with a time step of 0.002 ps. GROMACS routines and the VMD program were used to assess the quality of MD trajectories and to perform structural analyses. Several parameters of the simulations (timescale, box dimensions, number of water molecules) are reported in Table S1.

#### Radical scavenger activity (RSA) assay

A TEMPO water solution (5.0·10^–4^ mol/L) was prepared dissolving the radical powder in water. UV–Vis spectra of TEMPO alone or containing 25 μg of PEG6-Y4 (heated in stove for 2 or 4 h) were recorded after 30 min of incubation at room temperature.

#### Fluorescence studies

Fluorescence measurements were carried out at room temperature with a spectrofluorophotometer Jasco (Model FP-750) and the sample was allocated in a quartz cell with 1.0 cm path length. The others setting are: excitation and emission bandwidths = 5 nm; recording speed = 125 nm/min and automatic selection of the time constant. Emission spectra of PEG6-Y4 were recorded on the peptide in water solutions at several concentrations between 0.05 and 0.80 mg/mL by exciting the sample at λ_ex_ = 276 nm. Fluorescence measurements aimed to the CAC determination were performed as previously reported^30^. Photoluminescence (PL) measurements were carried out using the same instrumental parameters exciting the peptide assemblies (concentration before heating is 5.0 mg/mL; concentration after heating is 5.0 mg/mL) at various wavelengths in the range between 370 and 600 nm.

#### Fluorescence microscopy and photostability study

Heated PEG6-Y4 were dissolved in MeOH at a concentration of 5.0 mg/mL. 10µL of this solution were drop-casted on a clean coverslip glass, dried and imaged with fluorescence microscopies. Immunofluorescence images were taken with a Leica DFC320 video-camera (Leica, Milan, Italy) connected to a Leica DMRB microscope equipped with a 10 X and 40 X objectives and the Image J Software (National Institutes of Health, Bethesda, MD) was used for analysis. The photostability of Tyr based nanospheres was assessed on the sample heated for 4 h, dissolved in MeOH and drop-casted on glass. The sample was left under continuous excitation (spectral region GFP, λ_exc_ = 488) for 180 min and fluorescence images were recorded at several time points (0, 15, 30, 45, 60, 75, 90, 120, 150 and 180 min). Fluorescence decay was reported as percentage respect to amount of the initial fluorescence.

## Supplementary Information

Supplementary Information.
