# Supplementary material for: Fabrication of fluorescent nanospheres by heating PEGylated tetratyrosine nanofibers
Source: Sci Rep. 2021 Jan 28;11:2470. doi: 10.1038/s41598-020-79396-7 (PMC7844296; doi:10.1038/s41598-020-79396-7)
Supplement: Supplementary file 1 — Supplementary Information. [file 41598_2020_79396_MOESM1_ESM.pdf]

Supplementary Information
for
Fabrication of fluorescent nanospheres by heating PEGylated tetratyrosine nanofibers

Enrico Gallo, Carlo Diaferia, Nicole Balasco, Teresa Sibillano, Valentina Roviello, Cinzia Giannini, Luigi Vitagliano, Giancarlo Morelli, Antonella Accardo*

*Address correspondence to this author:
Prof. A. Accardo,
Department of Pharmacy, Research Centre on Bioactive Peptides (CIRPeB), University of Naples "Federico II" and DFM Scarl, Via Mezzocannone 16, 80134-Naples, Italy.
e-mail: antonella.accardo@unina.it; Phone: +39-081-2532045; Fax: +39-081-2536642

Mr. E. Gallo
IRCCS SDN, Via E. Gianturco 113, 80143, Naples, Italy.

Dr. C. Diaferia, Prof. G. Morelli
Department of Pharmacy and Interuniversity Research Centre on Bioactive Peptides (CIRPeB), University of Naples "Federico II", via Mezzocannone 16, 80134 Naples, Italy.

Dr. N. Balasco, Dr. L. Vitagliano
Institute of Biostructures and Bioimaging (IBB), CNR, Via Mezzocannone, 16, 80134 Naples, Italy

Dr. T. Sibillano, Dr. C. Giannini
Institute of Crystallography (IC), CNR, Via Amendola 122, 70126 Bari (Italy)

Dr. V. Roviello
Department of Chemical, Materials and Industrial Production Engineering, DICMaPI, University of Naples "Federico II", Piazzale V. Tecchio 80, 80125, Naples, Italy.

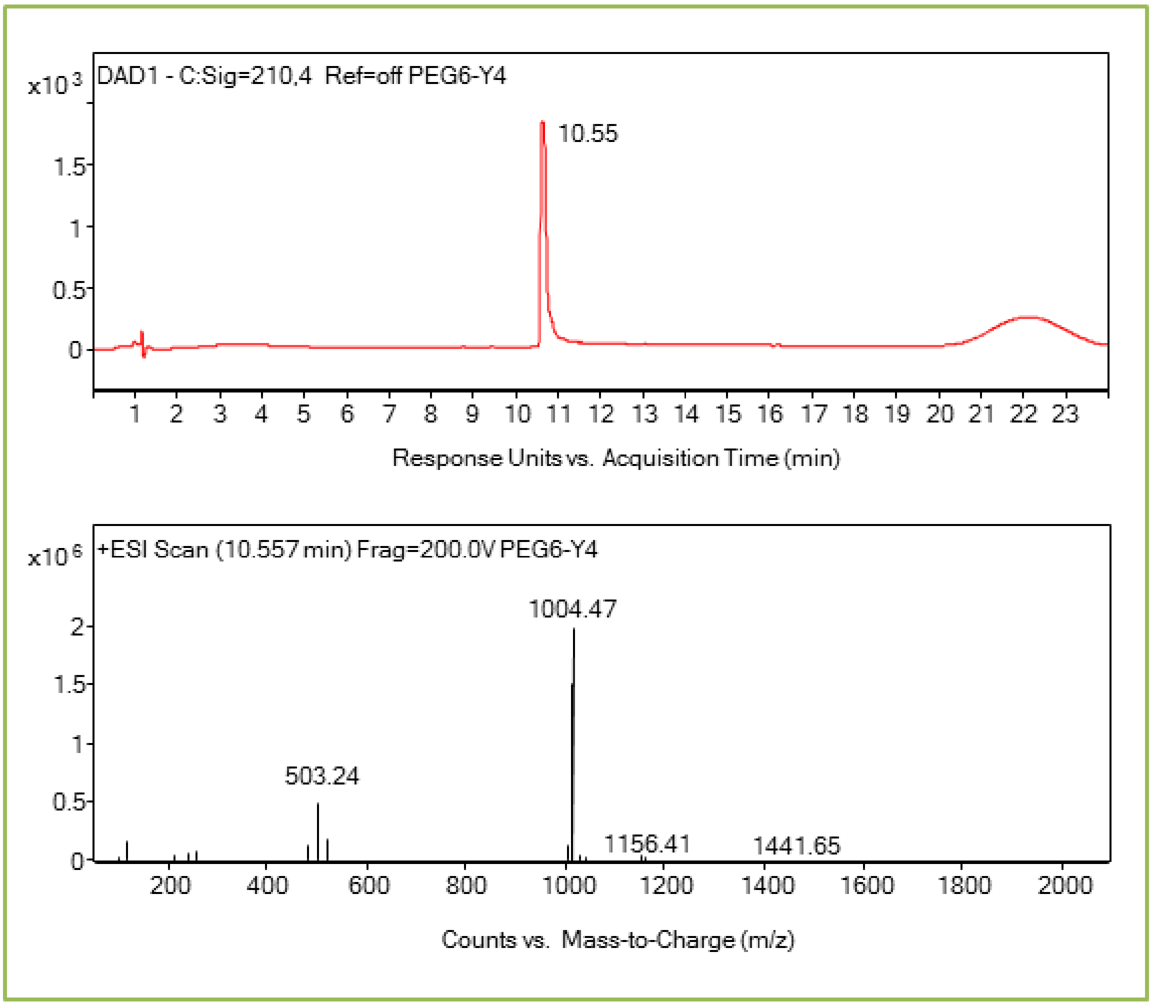

Figure S1: Chromatographic profile of PEG6-Y4 and the corresponding ESI mass spectrum.

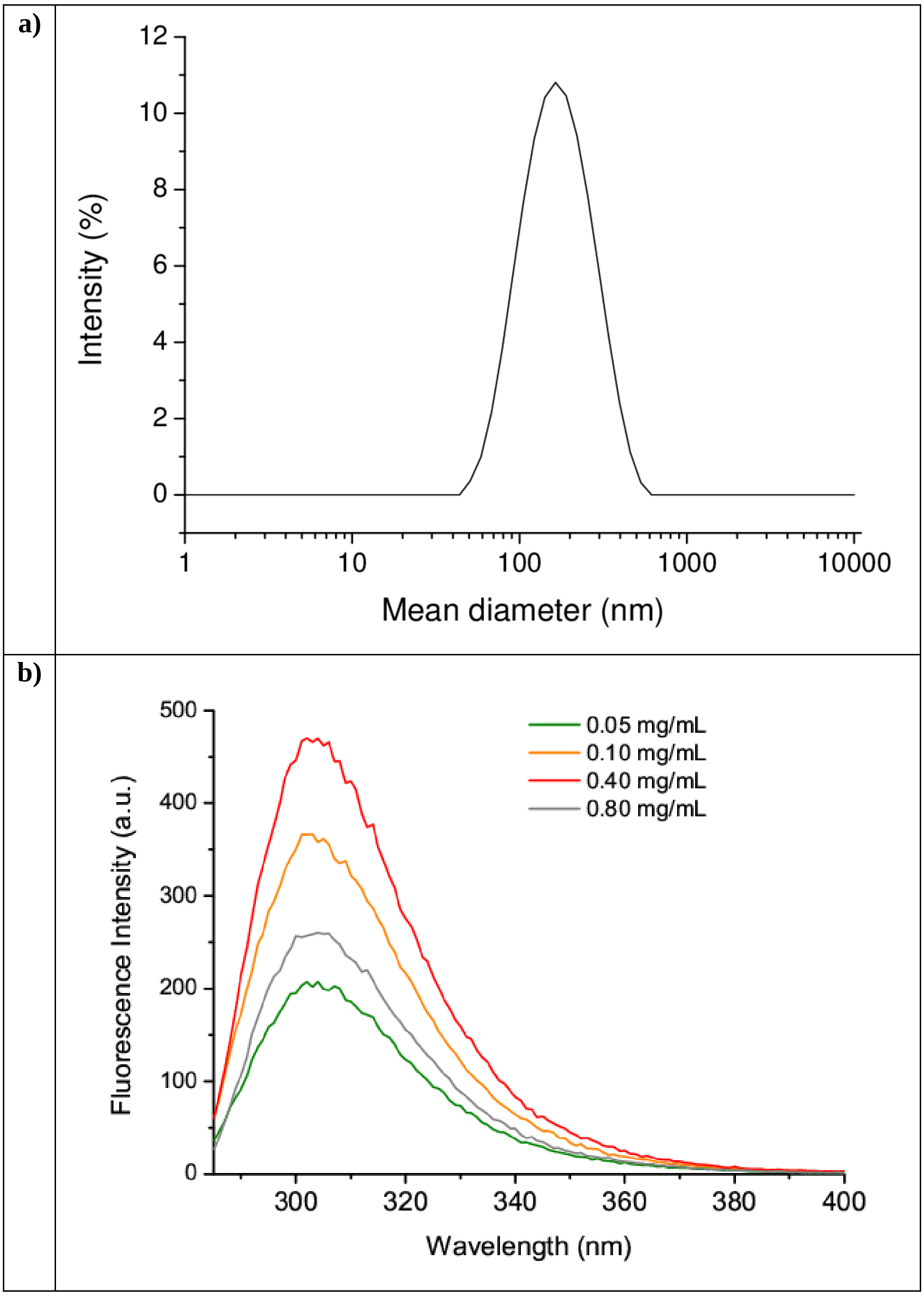

Figure S2: a) DLS intensity profile of PEG6-Y4. b) Fluorescence emission spectra of PEG6-Y4 excited at λ_{ex} = 276 nm in 0.05-0.8 mg/mL concentration range.

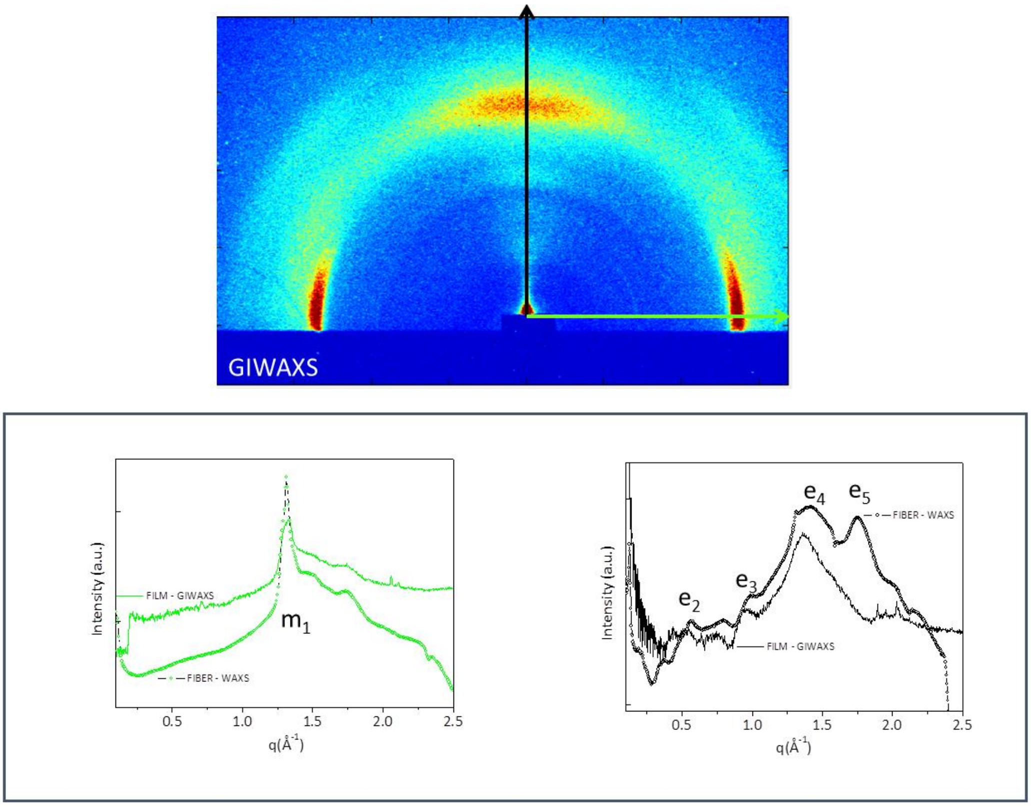

Figure S3: 2D GIWAXS image and corresponding 1D profiles integrated along meridional (in-plane direction, marked with a green arrow in the 2D GIWAXS image) and equatorial (out-of-plane direction, marked with a green arrow in the 2D GIWAXS image) directions. The 1D GIWAXS profiles are superimposed to the corresponding equatorial (black) and meridional (green) WAXS profiles, displayed in Figure 2a.

a)

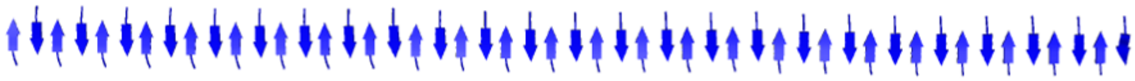

b)

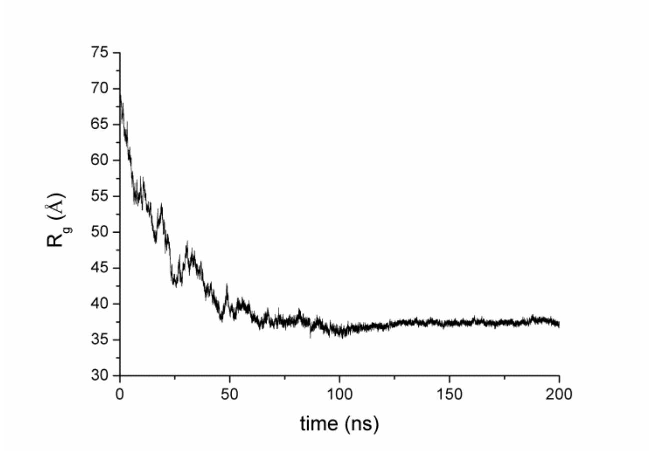

c)

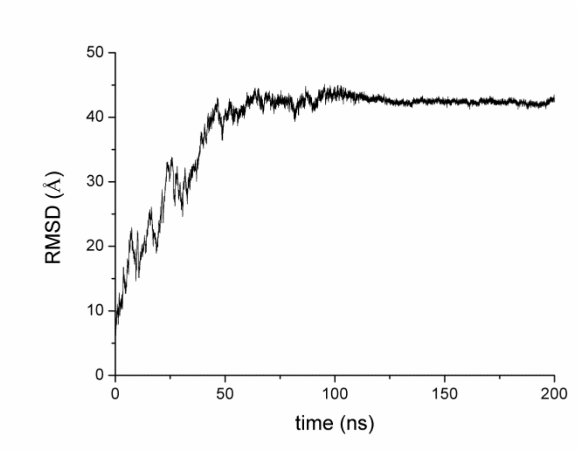

d)

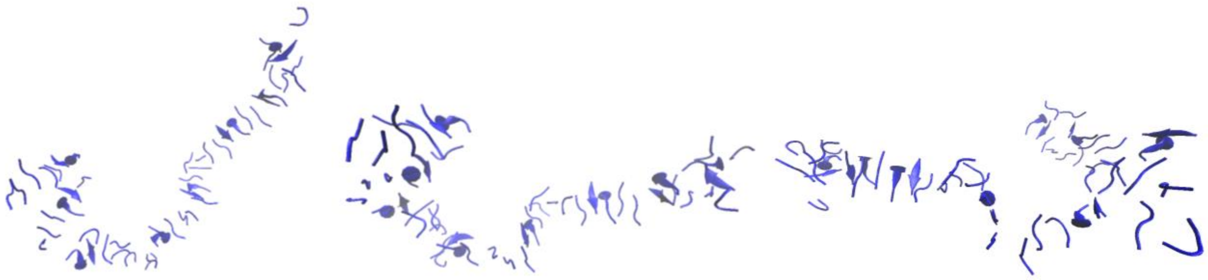

Figure S4: MD simulation of Y4_ST50_SH1. The starting model is reported in panel a). The gyration radius and the RMSD values of the trajectory structures *versus* the starting flat model are reported in panels b) and c), respectively. Representative examples of trajectory structures are reported in panel d).

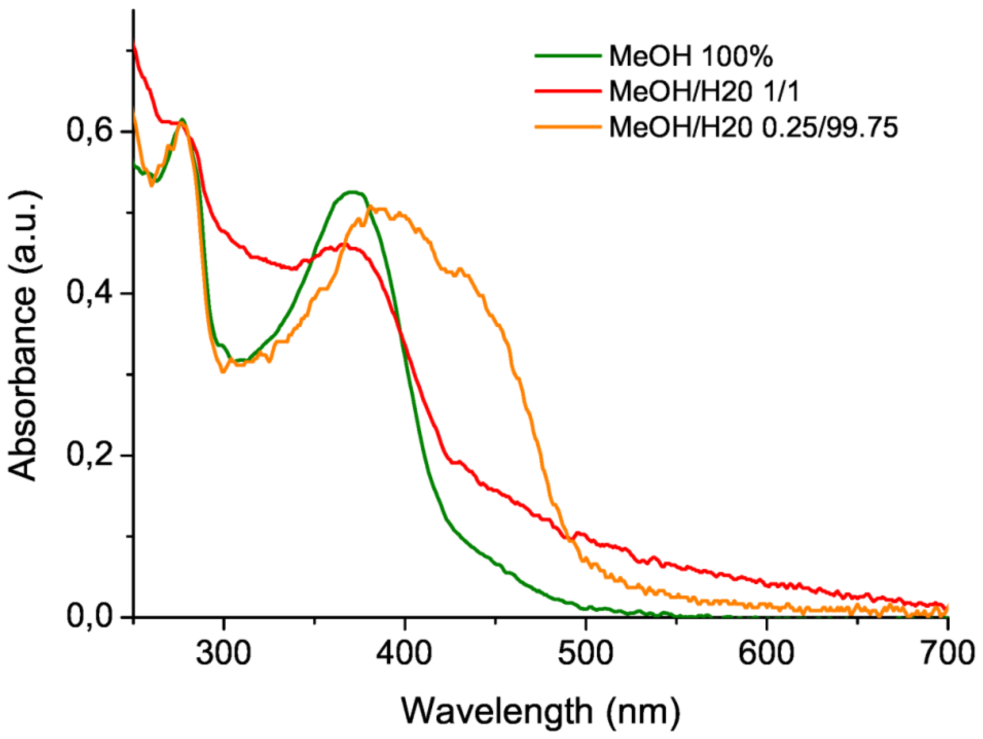

Figure S5: UV-Vis spectra of samples after thermal induced phase transition (4 hours) in MeOH, and in MeOH/H_2O mixture (1/1 and 0.25/99.75; v/v).

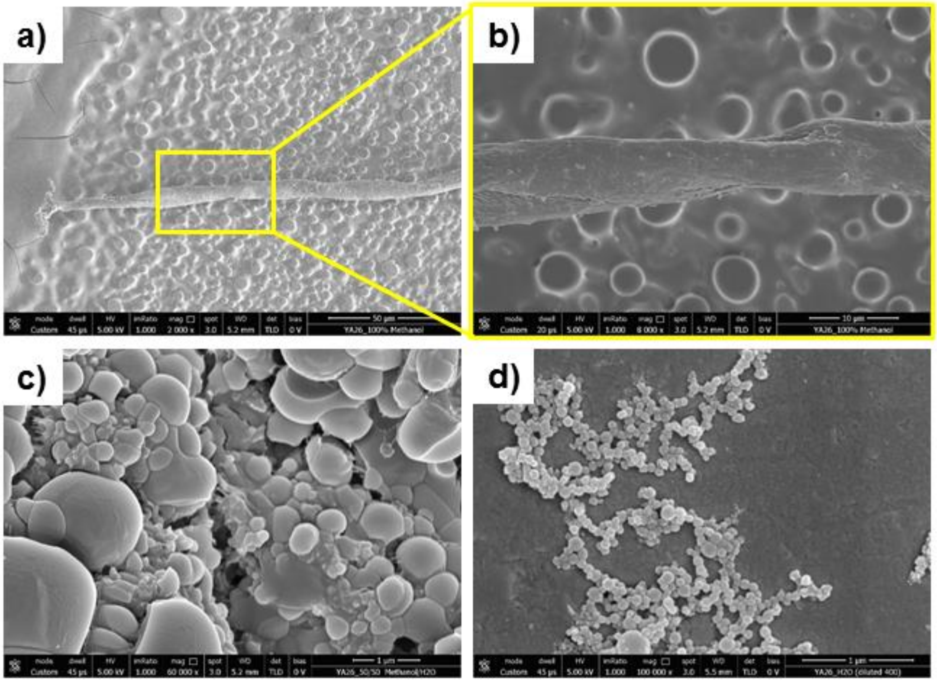

Figure S6: Selected SEM micrographs for self-assembled PEG6-Y4 nanostructures drop-casted after heating in stove for 2 hours and re-dissolved in MeOH (a, b), H_2O/MeOH (50/50, v/v) (c) and H_2O/MeOH (0.025/99.75, v/v) (d). Magnification and scale bar are 2000x, 50µm for (a); 8000x, 10µm for (b), 60000x, 1µm for (c) and 100000x, 1µm for (d).

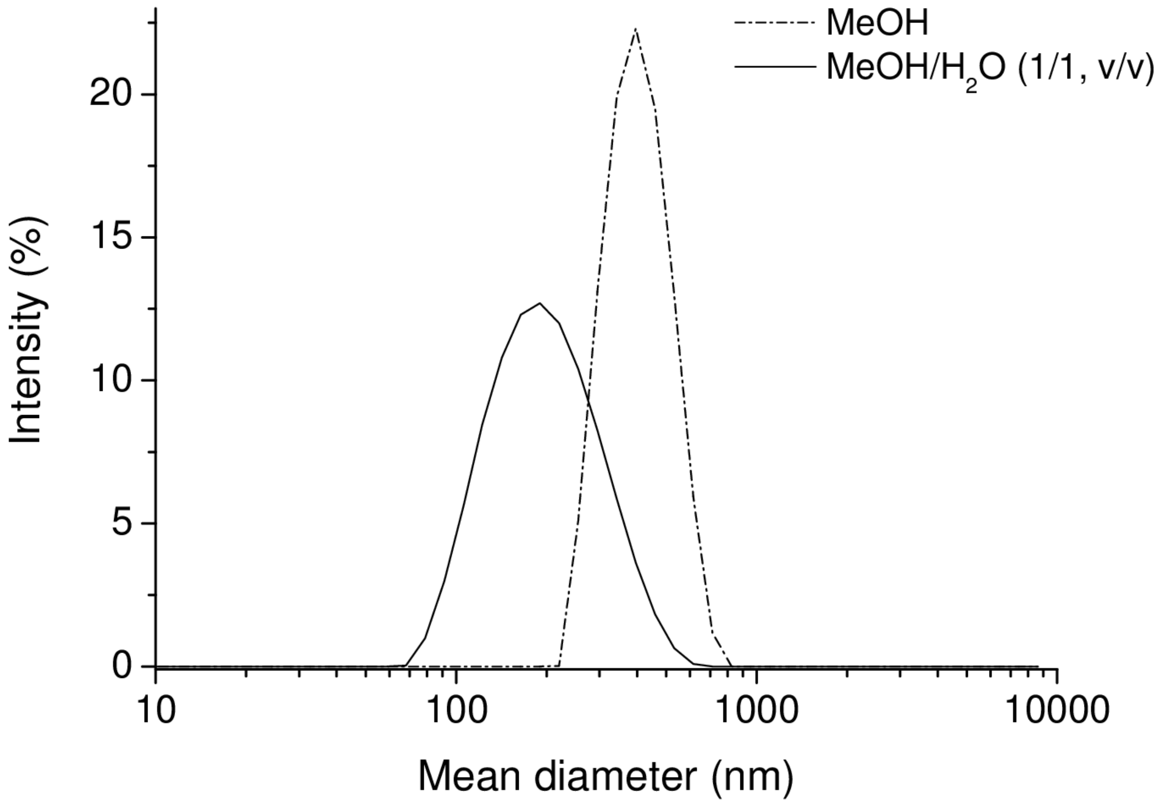

Figure S7: DLS intensity profiles of PEG6-Y4 after heating in stove for 4 hours and re-dissolved in 100% MeOH or in MeOH/H_2O (50/50, v/v) mixture.

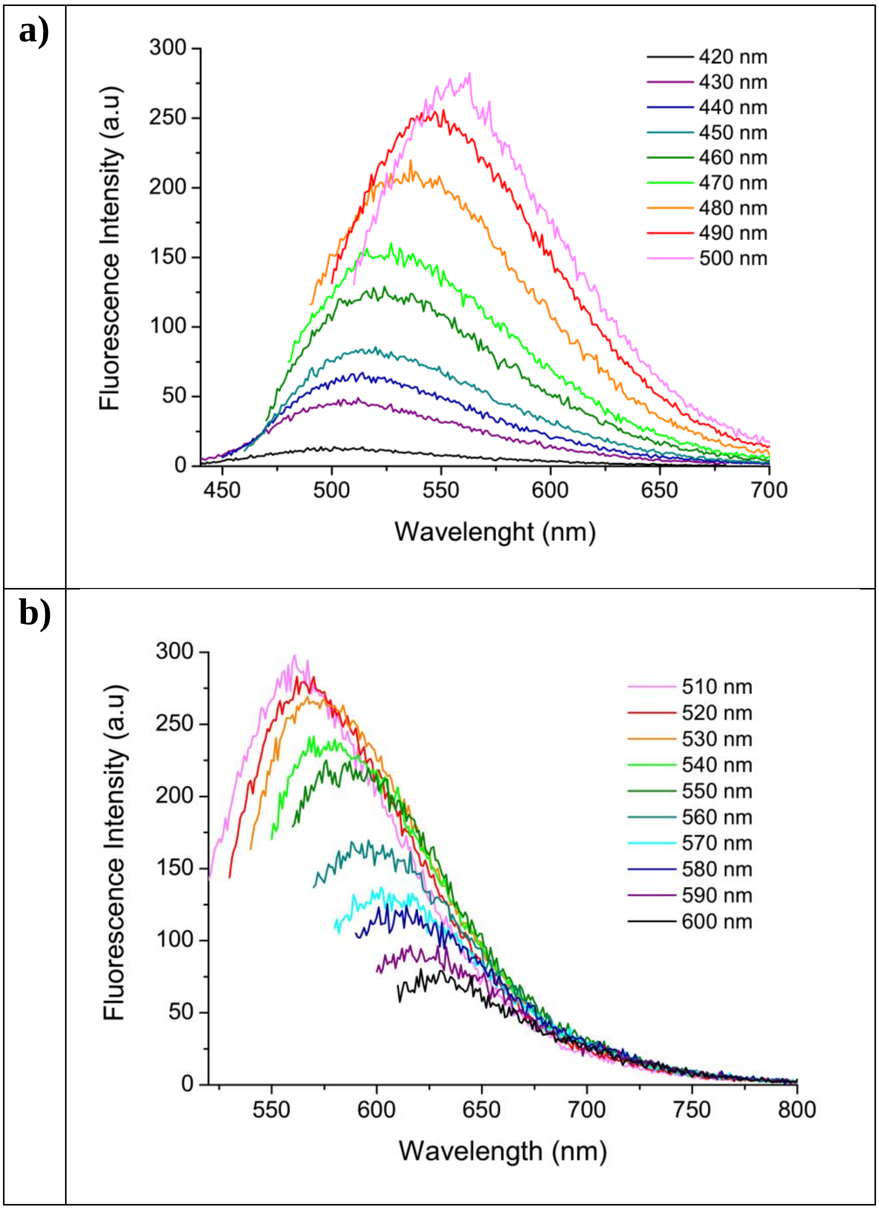

Figure S8: PL emission spectra of PEG6-Y4 assemblies after 4 hours of heating at 5.0 mg/mL in 100% MeOH upon excitation in the range 420 < λ_{exc} < 500 nm (a) and in the range 510 <λ_{exc}< 600 nm (b)

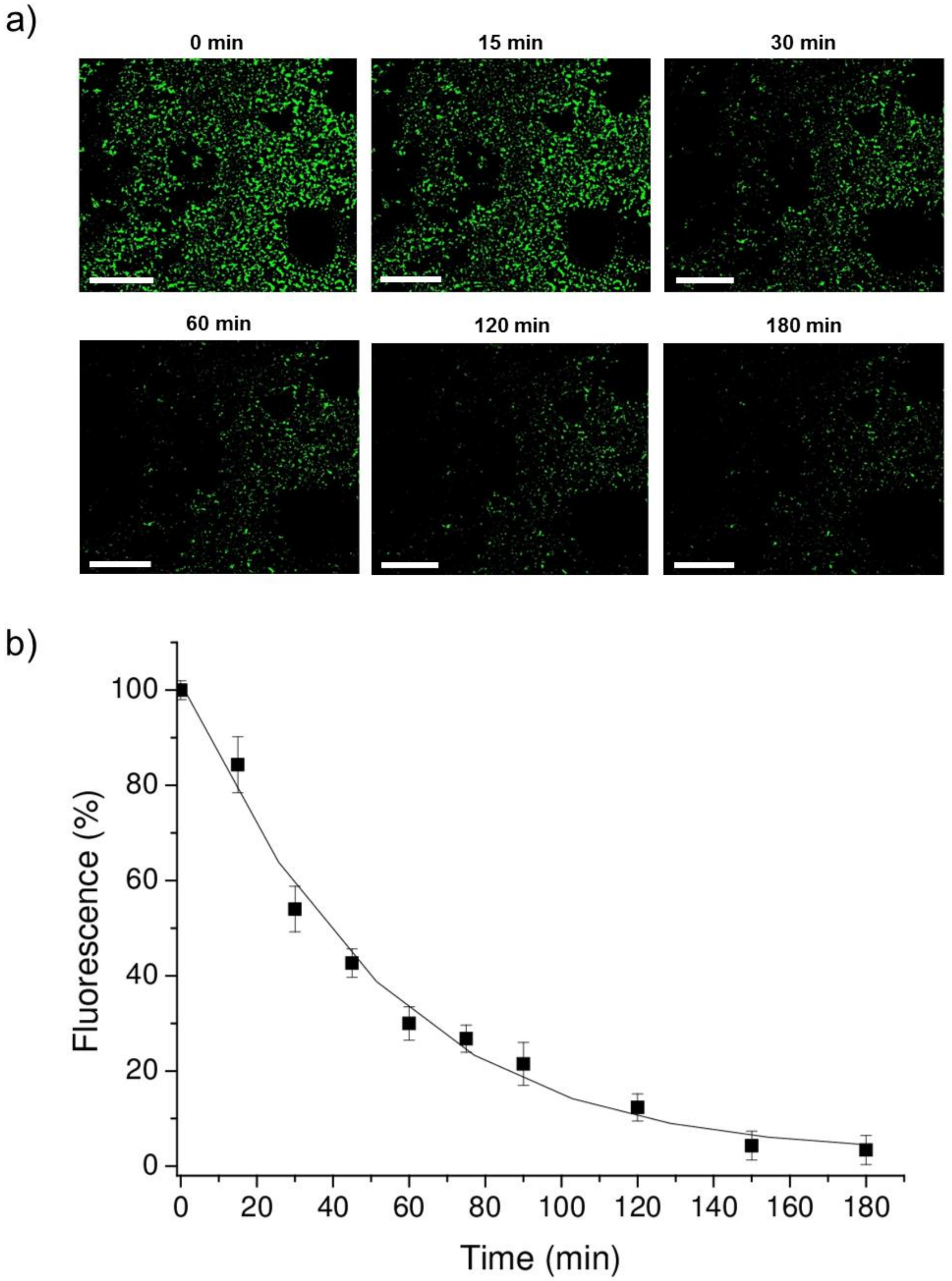

Figure S9: (a) Selected fluorescence microscopy images of the heated peptide solution (10 μL) drop-casted on glass slide and air-dried at room temperature. Images are obtained by exciting the sample in the spectral region of green fluorescent protein (GFP) and keeping the light on for 180 min. In figure are reported images at 0, 15, 30, 60, 120, 180 min. Scale bar is 50 μm. (b) Evaluation of the fluorescence intensity percentage decrease due to photoinstability phenomena.

Table S1. Parameters of the MD simulations.

System	Timescale (ns)	Box dimensions (nm^3)	Water molecules
Y4_ ST50_SH1	200	4.28 x 25.87 x 4.27	14147
Y4_ ST50_SH2	300	4.50 x 26.02 x 5.90	19702
